# Synergistic Effect of Organic Waste Products and Microbial Inocula on Iron and Zinc Biofortification in Cowpea [*Vigna unguiculata* (L.) Walp.]

**DOI:** 10.1002/fsn3.71375

**Published:** 2026-01-05

**Authors:** Emmanuel Noumsi‐Foamouhoue, Samuel Legros, Paula Fernandes, Hassna Founoune‐Mboup, Bassirou Diallo, Komi Assigbetsé, Aboubacry Kane, Frédéric Feder, Jean‐Michel Médoc

**Affiliations:** ^1^ CIRAD UPR Recyclage et Risque Montpellier France; ^2^ Laboratoire Mixte International IESOL ISRA‐IRD Bel‐Air Center Dakar Senegal; ^3^ Recyclage et Risque Université de Montpellier Montpellier France; ^4^ CIRAD UPR Recyclage et Risque Saint‐Denis France; ^5^ UPR HortSys CIRAD Montpellier France; ^6^ HortSys Université de Montpellier Montpellier France; ^7^ Laboratoire National de Recherches sur les Productions Végétales (LNRPV) Institut Sénégalais de Recherche Agriocole (ISRA) Dakar Senegal; ^8^ Institut de Recherche pour le Développement (IRD) Montpellier France; ^9^ FST, Département de Biologie Végétale, UCAD Dakar Senegal; ^10^ Laboratoire Commun de Microbiologie (LCM) IRD‐ISRA‐UCAD Bel‐Air Center Dakar Senegal

**Keywords:** agroecology, agronomic biofortification, local beneficial microorganisms, micronutrients, mycorrhizal fungi, organic waste products

## Abstract

Micronutrient deficiencies affect over 2 billion people worldwide, with iron (Fe) and zinc (Zn) deficiencies prevalent in Senegal. These deficiencies result from the low Fe and Zn contents in food crop products. This study examined the effects of micronutrient‐rich organic waste products (OWPs), in combination with local beneficial microorganisms and mycorrhizal fungi, on Fe and Zn concentrations in cowpea ( *Vigna unguiculata* ) grains and haulms. Cowpea trials were conducted over two contrasting consecutive seasons (wet and dry). A factorial block design with four replications was used. The main results showed significant increases in cowpea yield (up to 2.4‐fold for grains and 3.2‐fold for haulms), Fe concentrations (up to +48% in grains and +259% in haulms), and Zn concentrations (up to +28% in grains and +265% in haulms) with the application of OWP combined with microbial inocula (MIs), compared with those in the control. In addition, the observed effects on the yield and Fe and Zn concentrations depended on the type of OWPs and MIs used. These results validate our initial hypothesis regarding the significant increase in Fe and Zn content in cowpea grains and haulms with the combined application of OWPs, which supply micronutrients, and MIs that facilitate the solubilization and transfer of these micronutrients to the plant. Our findings provide novel insights into the agro‐biofortification of cowpeas and can be further developed to guide the selection of OWPs and MIs for use in agroecological biofortification systems.

## Graphical abstract

Agro‐biofortification using micronutrient‐rich organic waste products combined with local beneficial microorganisms and mycorrhizal fungi substantially enhanced iron and zinc concentrations in cowpea. Across contrasting seasons, this integrated approach increased Fe by up to 48% in grains and 259% in haulms, and Zn by up to 28% in grains and 265% in haulms while substantially enhancing yields. These results highlight a promising agroecological strategy to address widespread Fe and Zn deficiencies in human diets.

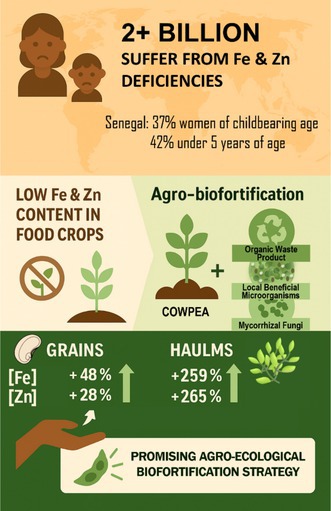

## 1 Introduction

Micronutrient deficiency, also known as hidden hunger, is a serious public health problem that coexists with two forms of malnutrition: under‐ and overnutrition. The 2024 report on the state of food security and nutrition in the world revealed that up to 2.8 billion people worldwide were at risk of micronutrient deficiency in 2022 (Haishan et al. 2025). Iron (Fe), zinc (Zn), iodine (I), and vitamin A are micronutrients whose deficiency in the diet is most frequent and damaging (Kam 2025; Ramzan et al. 2020). Fe and Zn deficiencies rank among the leading causes of disease in low‐income countries according to the World Health Organization (2002). This is the case in Senegal, where 37% of women of childbearing age and 42% of children under 5 years of age suffer from Fe/Zn deficiency (COSFAM 2022). Fe and Zn are essential for the proper functioning of living organisms. The main characteristic of these micronutrients is their extraordinary ability to bind proteins or organic coenzymes to attract substrate molecules and facilitate their conversion into specific end products (Carvalho et al. 2015).

Three widely recognized strategies for combating micronutrient deficiency include (i) supplementation with pharmaceutical preparations, (ii) food enrichment, and (iii) dietary diversification (Cakmak 2008; Li et al. 2017). Fe and Zn supplementation has proven useful in developing countries for rapidly improving the Fe and Zn status of deficient individuals; however, this strategy is relatively expensive and often poorly adhered to, particularly in the case of Fe, because of unpleasant side effects (Frossard et al. 2000). Food enrichment strategies use easily accessible and widely consumed carrier foods to prevent or correct deficiencies of one or more nutrients in large populations. However, the challenge is finding the right food approach in which the addition of micronutrients does not alter the stability, bioavailability, or organoleptic properties of the food, while maintaining accessibility and reasonable cost. Furthermore, the success of a fortification program requires strong political will and the involvement of all actors in the food chain. Dietary diversification can also be difficult in developing countries because of economic reasons, accessibility, and acceptance of diversified foods (Frossard et al. 2000). In Senegal, only one in five children is fed according to the criteria for dietary diversification (Ruel‐Bergeron 2017). In addition, rural diets, often monotonous, are mainly based on cereals, with limited consumption of animal‐source and Fe‐ and Zn‐rich foods (particularly fruits and vegetables) (Anderson et al. 2010). However, these common approaches to combating Fe and Zn deficiencies have not always been successful. Therefore, the identification of more efficient alternatives to combat micronutrient malnutrition is a key priority. Exploring how low‐income countries can increase access to micronutrient‐enriched foods has stimulated diverse research avenues in biofortification.

Biofortification is the process of increasing the micronutrient concentrations in crops through plant breeding, transgenic techniques, and/or agronomic practices (Bouis and Saltzman 2017). This is a potentially more sustainable strategy, and micronutrient‐enriched crops could reach more people than nutrient supplements or fortified foods and would also be cheaper (Frossard et al. 2000). Biofortification through agronomic practices includes mineral fertilization (application of chemical fertilizers to the soil and/or foliar spraying), organic fertilization (application of an organic material to the soil), and biological fertilization (application of a source material of beneficial microorganisms to the soil) (Khan et al. 2019; Çakmak and Kutman 2018; Chattha et al. 2017; Kamran et al. 2017; McGrath et al. 2012). Despite the considerable micronutrient gains in crop products via mineral fertilization reported in the literature (Parmar et al. 2023; Chattha et al. 2017; White et al. 2017), this biofortification practice is limited by the accessibility and cost of these fertilizers in rural areas. In addition, their use can lead to degradation of agricultural soils and groundwater pollution (Pahalvi et al. 2021; Robertson and Vitousek 2009). Several authors have demonstrated a significant increase in Fe and Zn in the harvested products of certain crops fertilized with organic waste products (OWPs) or microbial inocula (MIs). For instance, McGrath et al. (2012) showed an increase in Zn content in wheat ( *Triticum aestivum* ) grains fertilized with sewage sludge in England. Escobedo‐Monge et al. (2020) reported improved Fe content in potato ( *Solanum tuberosum* ) tubers fertilized with municipal solid waste compost in Spain. Kamran et al. (2017) reported an increase in Zn concentrations in wheat grains and stems fertilized with *Rhizobium*, *Pseudomonas*, *Enterobacter*, and *Pantoea* strains in Pakistan. Ali et al. (2022) reported improved grain quality and yield in wheat fertilized with *Trichoderma harzianum*. Marra et al. (2022) demonstrated that a *Trichoderma* strain enhanced amino acid and mineral contents in chickpea ( *Cicer arietinum* ).

To date, few studies have been conducted on the biofortification of local food crops in Senegal using Fe and Zn. Simultaneously, OWP and MI use is increasing in agricultural production systems in Senegal. Based on this observation, we propose the following hypotheses: (1) using OWPs significantly increases the micronutrient content of cowpeas [ *Vigna unguiculata* (L.) Walp.], which are widely consumed in rural areas, and (2) the addition of MIs enhances micronutrient gain from OWPs. This study aimed to assess the effects of OWPs in combination with MIs on Fe and Zn concentrations in cowpea haulms and grains.

## 2 Materials and Methods

### 2.1 Experimental Procedures

The experiment was conducted over two consecutive cropping seasons: rainy (June 2021–September 2021) and dry (November 2021–February 2022) seasons. The same treatments were applied to the same experimental units in both seasons to assess the residual effects of OWPs, local beneficial microorganisms (BMs), and mycorrhizal fungi (MF) applied in the first season on cowpea performance in the second season. In addition, repeated application of OWPs containing trace metals will enable the assessment of potential long‐term soil contamination risks. To ensure agronomic validity of the trial during the dry season, an irrigation system was implemented to compensate for the absence of rainfall, thereby providing controlled and comparable growing conditions. Furthermore, in tropical regions without winters, the variability between two successive seasons is comparable to that between two successive years during the same period. Therefore, the principle of exposing the treatments to contrasting climatic conditions was fulfilled. This approach is consistent with the mechanistic and time‐sensitive framework adapted to tropical double‐cropping systems. The trials were conducted at the Institut Sénégalais de Recherches Agricoles (ISRA) experimental station (13°45′29″ N, 15°47′12″ W) located in Nioro du Rip, south of the Peanut Basin. The meteorological data from the experimental stations during the study are shown in Figure S1. The soil in the area is classified as Lixisols (IUSS Working Group WRB 2015) with low organic matter content, cation‐exchange capacity (CEC), and total Zn levels (Table 1). Grains of the cowpea variety “*Thieye*” were provided by the ISRA seed service. Two types of OWPs were tested: poultry litter (PL, agricultural origin) and sewage sludge (SS, urban origin). Samples were collected from the Dakar region. Dry PL (droppings mixed with peanut shells) was obtained from a poultry house in Sangalkam. SS from the Dakar wastewater treatment process (methanization and total drying in open‐air beds) was sampled at the Cambérène Treatment Station. Two types of MI were tested in this study: BMs, a microbial consortia of bacteria and fungi obtained from the fermentation of forest litter collected south of the Peanut Basin and prepared with peanut shells following the procedure described by Noumsi‐Foamouhoue et al. (2023); MF *Rhizophagus irregularis* provided by the Laboratoire Commun de Microbiologie (LCM, IRD‐ISRA‐UCAD). The main chemical properties of OWPs and BMs are presented in Table 1. The microbial composition of BMs is shown in Table 2. OWPs, PL, and SS were selected because of their widespread use, widely contrasting nature and chemical composition, and high micronutrient contents, particularly Fe and Zn. Their selection was also based on their mineralization dynamics observed under controlled incubation conditions (Noumsi‐Foamouhoue et al. 2023). BMs were selected based on the diversity and abundance of their microbial communities, as well as their performance in promoting carbon and nitrogen mineralization from OWPs (Noumsi‐Foamouhoue et al. 2023). MFs and the cowpea variety “Thieye” were selected for their demonstrated performance in enhancing micronutrient bioavailability in cowpea biomass, as shown in greenhouse‐based bioavailability tests conducted as part of the OR4Food project.

**TABLE 1:** Main soil, organic waste product (OWP), and BM properties.

	Soil	PL	SS	BM
TOC (g 100 g^−1^ DM)	0.28	27.36	23.75	34.24
TON (g kg^−1^ DM)	0.20	65	25.50	9.70
N‐NO_3_ (mg kg^−1^ DM)	0.80	n.d.	n.d.	n.d.
N‐NH_4_ (mg kg^−1^ DM)	5.40	n.d.	n.d.	n.d.
C/N	13	4.21	9.31	35.30
Total P (mg kg^−1^ DM)	65	12,941	9274	580
Assim. P (mg kg^−1^ DM)	7	1141	196	192
CEC (cmol(+) kg^−1^ DM)	1.47	n.d.	n.d.	n.d.
WC (g 100 g^−1^)	3.32	8.12	3.74	59
pH_(H2O)_	6.67	7.57	6.98	7.28
Fe (mg kg^−1^ DM)	4728	6210	15,724	4.34
Zn (mg kg^−1^ DM)	< 50	326	364	20
Sand (g 100 g^−1^ DM)	83.50	n.d.	n.d.	n.d.
Silt (g 100 g^−1^ DM)	9.50	n.d.	n.d.	n.d.
Clay (g 100 g^−1^ DM)	5.40	n.d.	n.d.	n.d.

*Note:* Data presented for soil, OWP, and BM, dried at 105°C.

Abbreviations: BM, local beneficial microorganisms; CEC, exchange capacity; n.d., not determined; PL, poultry litter; SS, sewage sludge; TOC, total organic carbon; TON, total organic nitrogen; WC, water content.

**TABLE 2:** Diversity and abundance of local BMs (Noumsi‐Foamouhoue et al. 2023).

	Groups	Genus	BM
Bacteria (% OTU)	*Proteobacteria*	*Acetobacter*	28.45
		*Acinetobacter*	0.01
		*Pseudomonas*	0.01
		*Enterobacter*	0.34
		*Pantoea*	0.13
		*Rhizobium*	0.05
		*Methylobacterium*	0.01
		*Stenotrophomonas*	0.01
		*Burkholderia*	0.01
		*Bradyrhizobium*	0.03
		*Mesorhizobium*	0.00
		*Devosia*	0.03
		*Pseudoaminobacter*	0.00
	*Actinobacteria*	*Mycobacterium*	0.01
	*Firmicutes*	*Lactobacillus*	40.00
		*Bacillus*	5.35
		*Weissella*	5.08
		*Paenibacillus*	0.27
		*Clostridium*	8.01
		*Lysinibacillus*	0.83
		*Rummeliibacillus*	2.92
		*Pediococcus*	0.74
		*Virgibacillus*	0.01
	*Bacteroidota*	*Sphingobacterium*	0.02
		*Chryseobacterium*	0.00
Fungi (% OTU)	*Ascomycota*	*Neoascochyta*	33.75
		*Ascochyta*	3.59
		*Myrothecium*	2.13
		*Cladosporium*	0.01
		*Colletotrichum*	0.05
	*Basidiomycota*	*Yueomyces*	2.11

Abbreviations: BM, beneficial microorganisms; OTU, operational taxonomic units.

### 2.2 Trial Establishment and Maintenance

A randomized block design was used with four repetitions. The total plot size was 23 m × 13 m. Ten treatments were applied according to the above objectives (Table 3). An experimental plot covering 299 m^2^ was initially plowed using a disk plow. After pre‐irrigating the soil, OWPs were regularly incorporated into the corresponding elementary plots at an estimated depth of 20 to 30 cm, at rates of 2.07 t dry matter ha^−1^ (equivalent to 2.25 t crude matter ha^−1^) for PL and 6.11 t dry matter ha^−1^ (equivalent to 6.35 t crude matter ha^−1^) for SS. These OWP rates were determined according to the N, P, and K requirements of cowpeas (Gret, Cirad, Ministère français des Affaires étrangères 2002) and the fertilizer equivalency coefficient (Leclerc 2001) (Table S1). The quantities of Fe and Zn provided by OWPs are listed in Table S2. Solid BM inoculum was incorporated into the corresponding elementary plots at a dose of 15 t dry matter ha^−1^ (equivalent to 36.54 t crude matter ha^−1^). The MF inoculum was introduced into the seedbeds of the corresponding elementary plots at a rate of 1.88 t ha^−1^. Cowpea was sown at a density of 140 kg ha^−1^, on June 3, 2021, for season 1, and on November 1, 2021, for season 2. The cowpeas were thinned, leaving two plants per hole 14 days after sowing. For the chemical fortification treatment Fe_Zn, a solution of FeSO_4_·7H_2_O and ZnSO_4_·7H_2_O was prepared at concentrations of 0.81 and 5 g L^−1^, respectively. These solutions were applied by foliar spraying at the early growth and pod‐filling stages, with total application rates corresponding to 0.57 kg ha^−1^ of FeSO_4_·7H_2_O and 3.5 kg ha^−1^ of ZnSO_4_·7H_2_O (Chattha et al. 2017; Godsey et al. 2003). Chemical supplements (Table S1) were applied to the corresponding elementary plots during the early growth (phosphate fertilizer) and flowering (potassium fertilizer) stages. Liquid BM inoculum (prepared from solid BM inoculum and fermented anaerobically for 15 days) was applied to the corresponding elementary plots, plants (2 mL m^−2^ every 7 days), and soil (10 mL m^−2^ every 14 days). Sprinkler irrigation was applied to the entire plot (Image S1) in the absence of rain. Cowpeas were harvested on September 29, 2021, for season 1 and on February 27, 2022, for season 2.

**TABLE 3:** Treatment factor modalities.

Treatments	Chemical fortification	OWP		MI	
		PL	SS	BM	MF
Control					
Fe_Zn	x				
PL		x			
SS			x		
PL_BM		x		x	
SS_BM			x	x	
PL_MF		x			x
SS_MF			x		x
PL_BM_MF		x		x	x

Abbreviations: BM, local beneficial microorganisms; MF, mycorrhizal fungi; MI, microbial inocula; OWP, organic waste product; PL, poultry litter; SS, sewage sludge.

### 2.3 Sample Preparation and Analysis

After harvesting, the dry weights (65°C for 72 h) of cowpea grains and haulms were assessed by weighing (0.01 g scale). Some cowpea samples (washed and dried haulms and dried grains) were ground using a ceramic grinder to avoid metal contamination. The ground plant samples were stored in polypropylene pots at a temperature between 20°C and 25°C. Fe and Zn concentrations in the grains and haulms were determined using extraction with ultra‐pure 1 M HNO_3_ and quantified using microwave plasma atomic emission spectrometry (MP‐AES 4200, Agilent Technologies, Santa Clara, CA, USA). The total phosphorus content was determined using a continuous‐flow analyzer (AutoAnalyzer 3; SEAL Analytical, Norderstedt, Germany). Assimilable phosphorus was measured using the Murphy–Riley method. Total carbon and nitrogen were analyzed using a Thermo Finnigan Flash EA 1112 elemental analyzer (Thermo Fisher Scientific, Waltham, MA, USA). Nitrate nitrogen (N‐${\mathrm{NO}}_{3}^{-}$) and ammonium nitrogen (N‐${\mathrm{NH}}_{4}^{+}$) were extracted with 1 M KCl and analyzed using a SEAL Technicon colorimetric system (Porvair Sciences, Wrexham, UK). CEC was determined following extraction with 1 M KNO_3_ and quantified using atomic absorption spectrometry (AA220 FS Flame Spectrometer; Agilent Technologies). Soil pH was measured in a soil‐to‐water suspension at a 1:5 ratio. Soil texture (sand, silt, and clay fractions) was assessed after organic matter removal with H_2_O_2_ and particle dispersion using Na_4_P_2_O_7_.

### 2.4 Calculation and Statistical Analysis

The Fe and Zn extraction yields per hectare from the grains and haulms were calculated as follows:

$$Y_{\mathrm{MN}\left(x\right)}\left[\mathrm{kg}\;{\mathrm{ha}}^{-1}\right]=\frac{C_{\mathrm{MN}\left(x\right)}\left[\mathrm{mg}\;{\mathrm{kg}}^{-1}\right]\times {\mathrm{DW}}_{\left(x\right)}\left[t\;{\mathrm{ha}}^{-1}\right]}{1000}$$

where *Y*_MN(*x*)_ is the micronutrient yield (Fe or Zn) in sample *x* (grains or haulms), *C*_MN(*x*)_ is the micronutrient concentration in sample *x*, and DW_(*x*)_ is the dry weight of sample *x*.

Statistical analyses were performed using R software version 4.4.2 (R Foundation for Statistical Computing, Vienna, Austria). Data normality was verified using the Kolmogorov–Smirnov test, and the homogeneity of variances was checked using Levene's test. The main effects of seasons and treatments, as well as their interactions, were analyzed using two‐way ANOVA. The Scheirer–Rayleigh test was applied to non‐normally distributed data. Multiple comparisons of treatments within each season were conducted using the Newman–Keuls test. The Kruskal–Wallis test was applied to non‐normally distributed data. All tests were performed at a 5% significance level. Figures display data distributions using boxplots, where the central line represents the median, box limits represent the first and third quartiles (Q1 and Q3), and whiskers extend to values within 1.5‐fold the interquartile range. Outliers beyond this range are excluded from the display.

## 3 Results

### 3.1 Cowpea Yields

**FIGURE 1:**
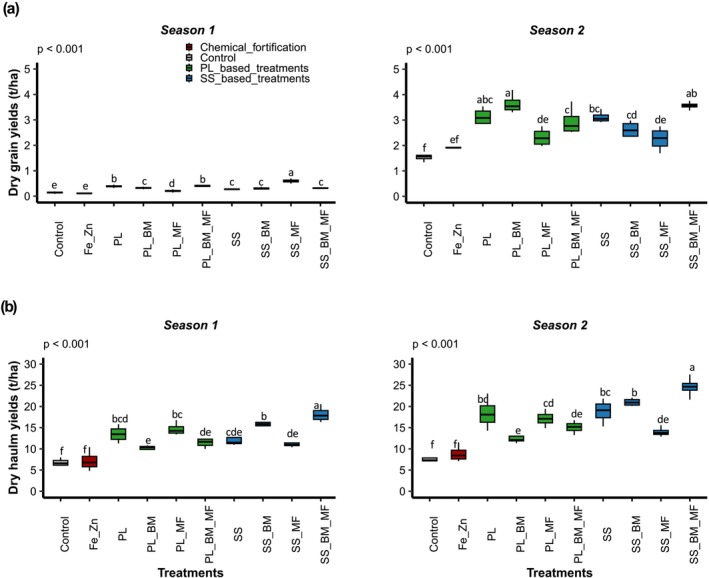
Dry grain (a) and haulm yields (b) in seasons 1 (June 2021–September 2021) and 2 (November 2021–February 2022) for the following treatments: Control (soil without input), Fe_Zn (chemical fortification with FeSO_4_·7H_2_O + ZnSO_4_·7H_2_O), PL (poultry litter), PL_BM (poultry litter + local beneficial microorganisms), PL_MF (poultry litter + mycorrhizal fungi), PL_BM_MF (poultry litter + local beneficial microorganisms + mycorrhizal fungi), SS (sewage sludge), SS_BM (poultry litter + local beneficial microorganisms), SS_MF (poultry litter + mycorrhizal fungi), SS_BM_MF (poultry litter + local beneficial microorganisms + mycorrhizal fungi). Groups with different letters significantly differ at *p* < 0.05.

The two factors, treatment type and sowing season, significantly affected cowpea dry grain and dry haulm yields (*p* < 0.001; Table 4). The significant interaction between seasons and treatments (*p* < 0.05) emphasized the importance of comparing the efficacy of treatments within each season (Figure 1).

**TABLE 4:** Effects of seasons, treatments, and season × treatment interaction on cowpea dry grain and dry haulm yields.

Plant part	Variation source	Modalities	Mean ± SD (t ha^−1^)	*F*	Pr
Dry grains	Seasons	Season 1	0.31 ± 0.14 b	2043	< 0.001
		Season 2	2.71 ± 0.73 a		
	Treatments			20.60	< 0.001
	Season × treatment			15.48	< 0.001
Dry haulms	Seasons	Season 1	12.14 ± 3.72 b	80.15	< 0.001
		Season 2	15.84 ± 5.37 a		
	Treatments			45.11	< 0.001
	Season × treatment			2.19	< 0.05

*Note:* Values followed by different letters in the same group are significantly different at the 5% threshold.

Abbreviations: *F*, Fisher statistic, value of differences between means; Pr, *p*‐value, significance level associated with *F*; SD, standard deviation.

During season 1, OWPs positively affected grain yield. The PL and SS treatments resulted in significantly higher grain yields than those in the control or Fe_Zn treatments (Figure 1a). Grain yield in the PL treatment was significantly higher than that in the SS treatment (Figure 1a). MI exhibited contrasting effects on grain yield. In particular, the grain yields of PL_BM_MF were similar to those of PL, whereas those of PL_BM and PL_MF were significantly lower (Figure 1a). Additionally, an effect of MI type was observed. Specifically, the yield of PL_BM_MF was significantly higher than that of PL_BM and PL_MF (Figure 1a). OWPs also positively affected haulm yield. The PL and SS treatments resulted in significantly higher haulm yields than those of the control or Fe_Zn treatments (Figure 1b). MI also had contrasting effects on yield. Specifically, SS_BM significantly increased haulm yield compared with that of SS, whereas PL_BM significantly decreased haulm yields compared with those of PL (Figure 1b). In addition, MI type exhibited an effect. The haulm yield of SS_BM was significantly higher than that of SS_MF (Figure 1b). During season 2, results similar to those for season 1 were observed. Moreover, in the second season, the grain and haulm yields of SS_BM_MF were significantly higher than those of SS_BM and SS_MF (Figure 1a,b). Thus, the highest yields of dry grains (3.64 ± 0.39 t ha^−1^) and dry haulms (24.62 ± 2.41 t ha^−1^) were obtained with PL_BM and SS_BM_MF, respectively, in season 2 (Figure 1a,b).

### 3.2 Fe Concentrations

**FIGURE 2:**
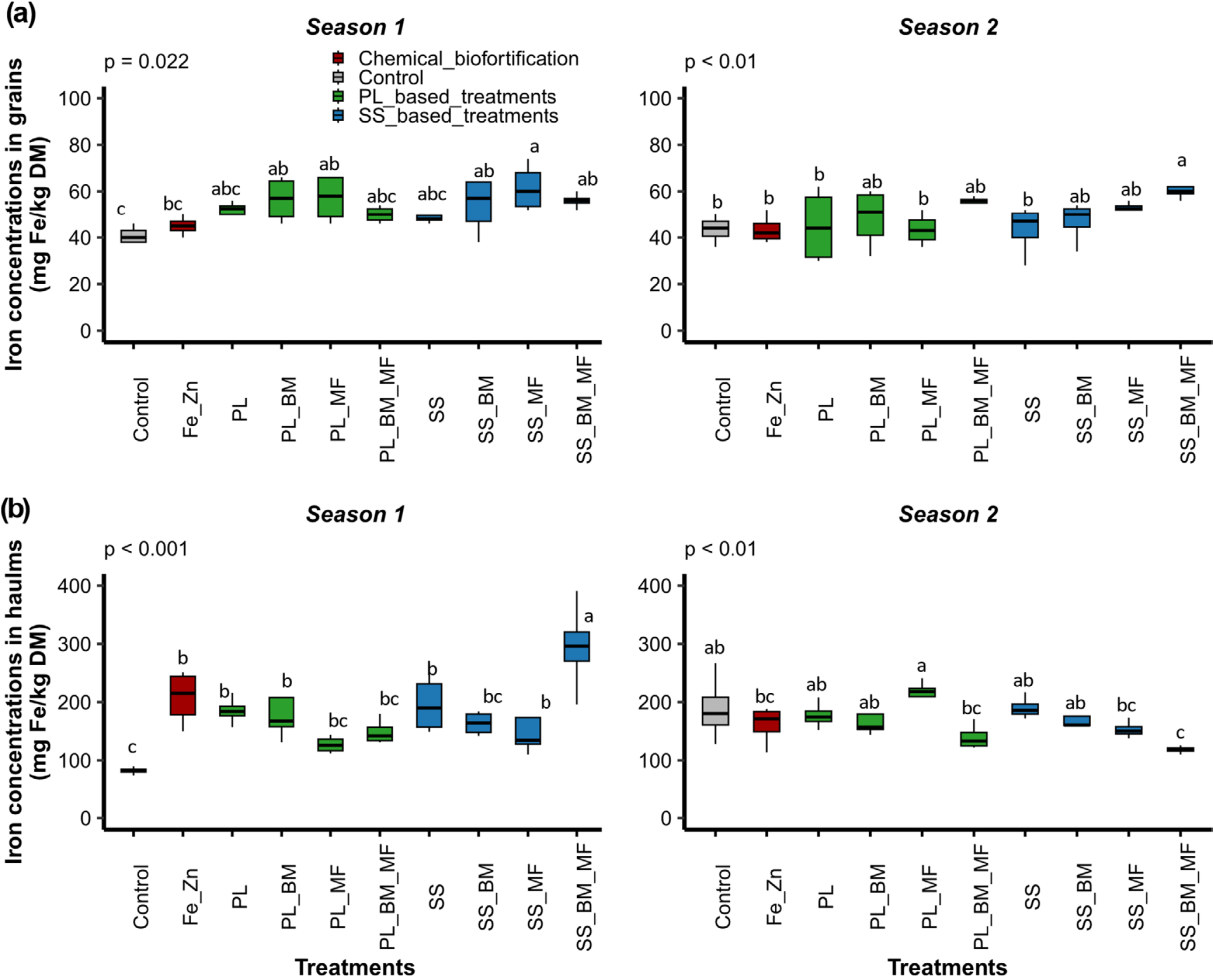
Iron concentrations in grains (a) and haulms (b) in seasons 1 (June 2021–September 2021) and 2 (November 2021–February 2022) for the following treatments: Control (soil without input), Fe_Zn (chemical fortification FeSO_4_·7H_2_O + ZnSO_4_·7H_2_O), PL (poultry litter), PL_BM (poultry litter + local beneficial microorganisms), PL_MF (poultry litter + mycorrhizal fungi), PL_BM_MF (poultry litter + local beneficial microorganisms + mycorrhizal fungi), SS (sewage sludge), SS_BM (poultry litter + local beneficial microorganisms), SS_MF (poultry litter + mycorrhizal fungi), SS_BM_MF (poultry litter + local beneficial microorganisms + mycorrhizal fungi). Groups with different letters significantly differ at *p* < 0.05.

Treatment type significantly influenced Fe concentrations in grains and haulms (*p* < 0.05; Table 5). Although no significant difference was observed in Fe concentrations in haulms between the two seasons, the significant interaction between seasons and treatments (*p* < 0.001) showed that the effect of treatments on Fe concentrations in haulms depended on the sowing season (Figure 2).

**TABLE 5:** Effects of seasons, treatments, and season × treatment interaction on iron concentrations in grains and haulms.

Parameter	Plant part	Variation source	Modalities	Mean ± SD	*F*	Pr
Iron content (mg Fe kg^−1^ DM)	Grains	Seasons	Season 1	52.13 ± 8.86 a	4.01	< 0.05
			Season 2	48.43 ± 9.82 b		
		Treatments			3.26	< 0.05
		Season × treatment			1.17	0.33
	Haulms	Seasons	Season 1	177.30 ± 70.99	0.65	0.42
			Season 2	169.53 ± 38.13		
		Treatments			2.08	< 0.05
		Season × treatment			6.27	< 0.001

*Note:* Values followed by different letters in the same group are significantly different at the 5% threshold.

Abbreviations: DM, dry matter; *F*, Fisher statistic, value of differences between means; Pr, *p*‐value, significance level associated with *F*; SD, standard deviation.

During season 1, the combination of OWPs and MI positively affected Fe concentrations in grains. All OWP and MI combinations significantly increased the Fe concentrations in grains compared with those in the control (Figure 2a). The exception was PL_BM_MF, which did not differ significantly from the control (Figure 2a). The highest Fe concentration in grains (61.5 ± 10.38 mg kg^−1^ DM) was obtained with SS_MF. OWPs also positively affected Fe concentrations in the haulms. The PL and SS groups showed significantly higher Fe concentrations than those in the controls (Figure 2b). Chemical fortification was also observed. Specifically, Fe_Zn displayed higher Fe concentrations in the haulms than those in the control (Figure 2b). Moreover, the combination of OWPs and MI positively affected haulm Fe concentrations. Fe concentrations in the haulms of SS_BM_MF were significantly higher than those in the SS (Figure 2b). The highest Fe concentration in haulms (295 ± 80 mg kg^−1^ DM) was obtained following SS_BM_MF treatment. Fe concentrations in the haulms of SS_BM_MF were significantly higher than in those of SS_BM and SS_MF (Figure 2b). During season 2, results comparable to those of season 1 were observed. Furthermore, during the second season, SS_BM_MF significantly reduced haulm Fe concentrations compared with those in SS (Figure 2b), while PL_MF exhibited the highest haulm Fe concentrations.

### 3.3 Fe Yield

**FIGURE 3:**
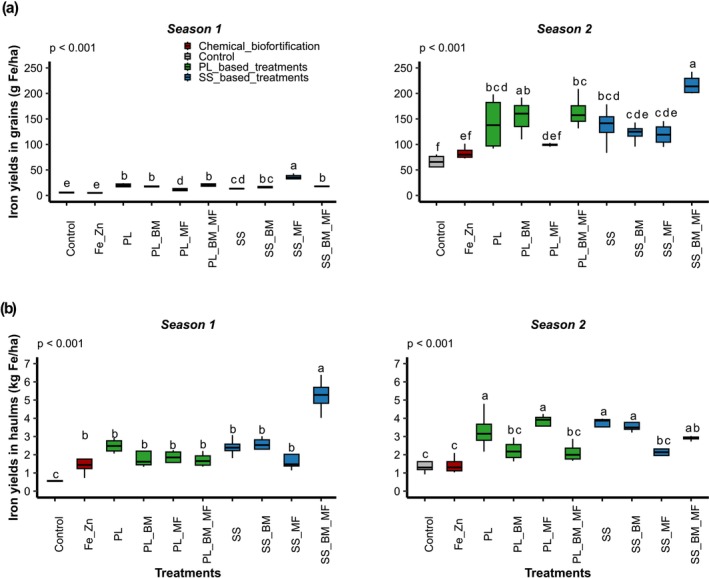
Iron yield in grains (a) and haulms (b) in seasons 1 (June 2021–September 2021) and 2 (November 2021–February 2022) for the following treatments: Control (soil without input), Fe_Zn (chemical fortification FeSO_4_·7H_2_O + ZnSO_4_·7H_2_O), PL (poultry litter), PL_BM (poultry litter + local beneficial microorganisms), PL_MF (poultry litter + mycorrhizal fungi), PL_BM_MF (poultry litter + local beneficial microorganisms + mycorrhizal fungi), SS (sewage sludge), SS_BM (poultry litter + local beneficial microorganisms), SS_MF (poultry litter + mycorrhizal fungi), SS_BM_MF (poultry litter + local beneficial microorganisms + mycorrhizal fungi). Groups with different letters significantly differ at *p* < 0.05.

Treatment and sowing season significantly influenced Fe yield in grains and haulms (Table 6; *p* < 0.01). The significant interactions between seasons and treatments (*p* < 0.001) emphasize the importance of comparing the efficacy of treatments across seasons (Figure 3).

**TABLE 6:** Effects of seasons, treatments, and season × treatment interaction on iron yields in grains and haulms.

Parameter	Plant part	Variation source	Modalities	Mean ± SD	*F*	Pr
Iron yield (g Fe ha^−1^)	Grains	Seasons	Season 1	16.41 ± 8.78 b	504	< 0.001
			Season 2	133 ± 52 a		
		Treatments			9.17	< 0.001
		Season × treatment			6.72	< 0.001
	Haulms	Seasons	Season 1	2240 ± 1306 b	8.43	< 0.01
			Season 2	2663 ± 1006 a		
		Treatments			15.12	< 0.001
		Season × treatment			5.98	< 0.001

*Note:* Values followed by different letters in the same group are significantly different at the 5% threshold.

Abbreviations: DM, dry matter; *F*, Fisher statistic, value of differences between means; Pr, *p*‐value, significance level associated with *F*; SD, standard deviation.

During season 1, OWPs positively affected grain Fe yield. The PL and SS treatments resulted in significantly higher grain Fe yields than those in the control and Fe_Zn treatments (Figure 3a). Additionally, OWP type exhibited an effect. In particular, PL showed significantly higher grain Fe yield than did SS (Figure 3a). The combination of OWPs and MI had contrasting effects on grain Fe yield. Specifically, the Fe yield in SS_MF grains was significantly higher than that in SS grains, whereas that in PL_MF grains was significantly lower than that in PL grains (Figure 3a). For haulms, the combination of OWPs and MI positively influenced Fe yield during season 1. SS_BM_MF displayed the highest Fe yield (5.24 ± 0.98 kg ha^−1^), which was significantly higher than that of SS (Figure 3b). Chemical fortification exhibited an effect. Notably, Fe_Zn exhibited higher haulm Fe yields than in the control (Figure 3b). The results for season 2 were comparable to those of season 1. Furthermore, in season 2, specific combinations of OWPs and MI led to reduced haulm Fe yield. In particular, SS_MF exhibited lower haulm Fe yields than did SS (Figure 3b), while the highest grain Fe yield was obtained with SS_BM_MF (218 ± 20 g ha^−1^) (Figure 3a).

### 3.4 Zn Concentrations

**FIGURE 4:**
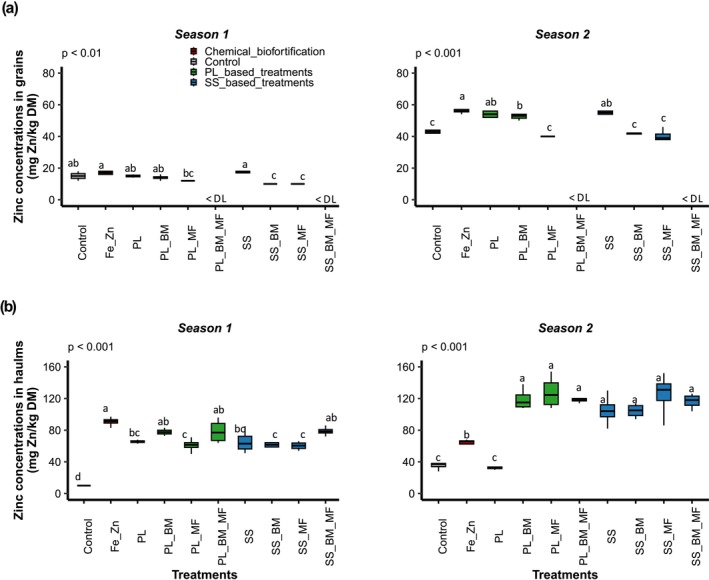
Zinc concentrations in grains (a) and haulms (b) in seasons 1 (June 2021–September 2021) and 2 (November 2021–February 2022) for the following treatments: Control (soil without input), Fe_Zn (chemical fortification FeSO_4_·7H_2_O + ZnSO_4_·7H_2_O), PL (poultry litter), PL_BM (poultry litter + local beneficial microorganisms), PL_MF (poultry litter + mycorrhizal fungi), PL_BM_MF (poultry litter + local beneficial microorganisms + mycorrhizal fungi), SS (sewage sludge), SS_BM (poultry litter + local beneficial microorganisms), SS_MF (poultry litter + mycorrhizal fungi), SS_BM_MF (poultry litter + local beneficial microorganisms + mycorrhizal fungi), DL [detection limit of the analysis spectrometer (10 ppm)]. Groups with different letters significantly differ at *p* < 0.05.

Sowing season significantly influenced grain and haulm Zn concentrations (Table 7, *p* < 0.001). The significant interactions between seasons and treatments (*p* < 0.001) highlight the need to compare the efficacy of treatments within each season (Figure 4).

**TABLE 7:** Effects of seasons, treatments, and season × treatment interaction on zinc concentrations in grains and haulms.

Parameter	Plant part	Variation source	Modalities	Mean ± SD	*F*	Pr
Zinc content (mg Zn kg^−1^ DM)	Grains	Seasons	Season 1	14.80 ± 2.91 b	3644	< 0.001
			Season 2	47.88 ± 7.09 a		
		Treatments			52.30	< 0.001
		Season × treatment			9.09	< 0.001
	Haulms	Seasons	Season 1	69.41 ± 16.03 b	85.85	< 0.001
			Season 2	94.78 ± 37.64 a		
		Treatments			27.41	< 0.001
		Season × treatment			15.98	< 0.001

*Note:* Values followed by different letters in the same group are significantly different at the 5% threshold.

Abbreviations: DM, dry matter; *F*, Fisher statistic, value of differences between means; Pr, *p*‐value, significance level associated with *F*; SD, standard deviation.

In season 1, similar to Fe, OWPs positively affected haulm Zn concentrations. The PL and SS treatments resulted in significantly higher haulm Zn concentrations than those in the control (Figure 4b). Chemical fortification also affected haulm Zn concentrations. The Fe_Zn treatment exhibited higher haulm Zn concentrations than those in the control (Figure 4b). MI had contrasting effects on Zn concentration. SS_BM and SS_MF showed significantly lower grain Zn concentrations than those in SS (Figure 4a). Moreover, haulm Zn concentrations in SS_BM_MF were significantly higher than those in SS_BM and SS_MF (Figure 3b). In season 2, OWPs positively influenced grain Zn concentrations. The PL, PL_BM, and SS treatments displayed significantly higher Zn concentrations in the grains than those in the control (Figure 4a). Chemical fortification also affected grain Zn concentrations. Fe_Zn treatment resulted in significantly higher grain Zn concentrations compared with those in the control (Figure 4a). Furthermore, OWP type affected haulm Zn concentrations. Specifically, SS treatment resulted in significantly higher haulm Zn concentrations than those in PL (Figure 4b). In the second season, both BM and MF positively affected haulm Zn concentrations. All combinations of OWP and MI significantly increased haulm Zn concentrations compared with those in PL. The haulm Zn concentrations of PL_MF were significantly higher than those of PL (Figure 4b). Thus, the highest grain Zn (56.5 ± 2.52 mg kg^−1^ DM) and haulm Zn (128 ± 21 mg kg^−1^ DM) concentrations were obtained with Fe_Zn and PL_MF, respectively, in season 2 (Figure 4a,b).

### 3.5 Zn Yields

**FIGURE 5:**
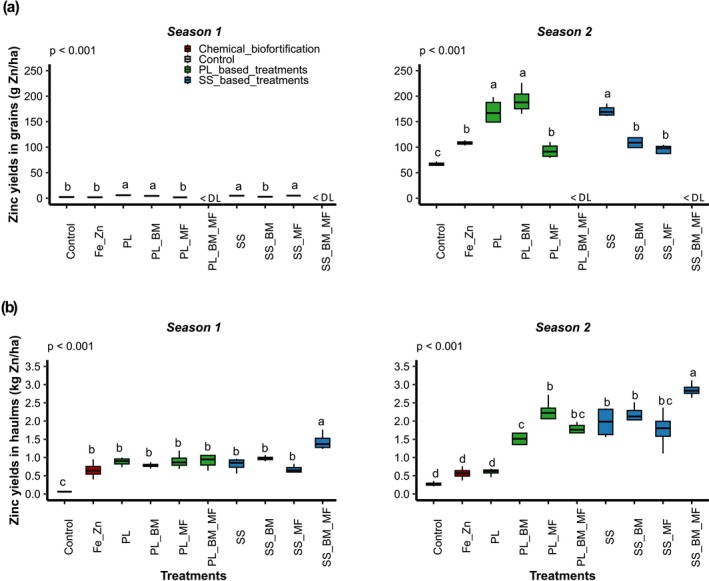
Zinc yields in grains (a) and haulms (b) in seasons 1 (June 2021–September 2021) and 2 (November 2021–February 2022) for the following treatments: Control (soil without input), Fe_Zn (chemical fortification FeSO_4_·7H_2_O + ZnSO_4_·7H_2_O), PL (poultry litter), PL_BM (poultry litter + local beneficial microorganisms), PL_MF (poultry litter + mycorrhizal fungi), PL_BM_MF (poultry litter + local beneficial microorganisms + mycorrhizal fungi), SS (sewage sludge), SS_BM (poultry litter + local beneficial microorganisms), SS_MF (poultry litter + mycorrhizal fungi), SS_BM_MF (poultry litter + local beneficial microorganisms + mycorrhizal fungi), DL [detection limit of the analysis spectrometer (10 ppm)]. Groups with different letters significantly differ at *p* < 0.05.

Sowing season significantly influenced Zn yields in grains and haulms (Table 8, *p* < 0.001). The significant interactions between seasons and treatments (*p* < 0.001) underscore the importance of evaluating treatment efficacy across seasons (Figure 5).

**TABLE 8:** Effects of seasons, treatments, and season × treatment interaction on zinc yields in grains and haulms.

Parameter	Plant part	Variation source	Modalities	Mean ± SD	*F*	Pr
Zinc yield (g Zn ha^−1^)	Grains	Seasons	Season 1	3.66 ± 1.54 b	1003	< 0.001
			Season 2	125 ± 46 a		
		Treatments			30.16	< 0.001
		Season × treatment			16.83	< 0.001
	Haulms	Seasons	Season 1	869 ± 295 b	164	< 0.001
			Season 2	1573 ± 844 a		
		Treatments			40.82	< 0.001
		Season × treatment			12	< 0.001

*Note:* Values followed by different letters in the same group are significantly different at the 5% threshold.

Abbreviations: DM, dry matter; *F*, Fisher statistic, value of differences between means; Pr, *p*‐value, significance level associated with *F*; SD, standard deviation.

In season 1, OWPs positively affected Zn yield. The PL and SS treatments showed significantly higher Zn yields in grains and haulms than those in the control (Figure 5a,b). Chemical fortification also exhibited effects on Zn yield. Notably, Fe_Zn treatment exhibited higher haulm Zn yields than those in the control (Figure 5b). MI demonstrated contrasting effects on Zn yield. Specifically, PL_MF showed lower grain Zn yields than those in PL (Figure 5a), whereas SS_BM_MF exhibited higher haulm Zn yields than those in SS (Figure 5b). In season 2, results similar to those for season 1 were observed. Additionally, during season 2, the OWP type affected Zn yield. SS treatment exhibited significantly higher haulm Zn yields than those in the PL treatment (Figure 5b). The type of MI also affected Zn yield. Specifically, PL_MF exhibited significantly higher haulm Zn yields than those in PL_BM (Figure 5b), and both combinations significantly increased Zn yields than those in PL. Thus, the highest Zn yields were obtained with PL_BM in grains (192 ± 26 g ha^−1^) and SS_BM_MF in haulms (2.86 ± 0.20 kg ha^−1^) during season 2 (Figure 5a,b).

## 4 Discussion

### 4.1 OWPs and MI Affect Cowpea Yields

The increase in grain and haulm yields with the application of OWPs observed in our study is consistent with literature and has been reported for a wide range of contexts, soil types, and OWP doses (Rodrigues et al. 2025; Ye et al. 2023; Krasilnikov et al. 2022; Sharma et al. 2019; Senesi 1989). For instance, Tagoe et al. (2008) showed an increase in the grain yields of cowpeas grown on loamy‐sandy soil fertilized with hen manure or treated urban organic waste compared with those in the unfertilized control. These results can be explained by the improvement in nutrient availability, water retention, and soil aeration with the addition of OWP, which contributed to improved plant growth and consequently increased crop yield (Gomgnimbou et al. 2019; Luciens 2014). Concerning the OWPs used in our study, PL showed, in some cases, better efficacy than that of SS in enhancing grain yields. This may be related to the different nature and composition of OWPs, in particular, the higher phosphorus and potassium contents of PL than those in SS (Table S1). Thus, the faster mineralization of PL, which has a low C/N ratio compared with that of SS (Table 1), released more phosphorus and potassium, which are beneficial to the plant at the tillering and flowering stages, respectively.

The addition of MI in combination with OWPs induced, in most cases, an increase in grain and haulm yields compared with OWP alone. These results are consistent with those reported previously (Lelamo 2025; Pandey et al. 2025; Sani et al. 2020; Bünemann et al. 2006). For instance, Yadav et al. (2019) also showed an improvement in the grain yield of cowpeas grown on a loamy‐sandy soil fertilized with a combination of OWPs (farmyard manure, vermicompost) and biofertilizers (*Rhizobium* and phosphorus‐solubilizing bacteria). The synergy between OWPs and MI could be explained by the microbial groups or genera present in the inocula. These have been identified in the literature as having the capacity to (i) mineralize carbon and nitrogen (*Firmicutes*, *Actinobacteria*, *Proteobacteria*, *Bacteroidota*, and *Ascomycota*) (Table 2), (ii) solubilize phosphorus (*Rhizophagus*, *Pantoea*, *Pseudomonas*) (Table 2), and (iii) increase the absorption surface of roots (*Rhizophagus*) (Cao et al. 2024; Ling et al. 2022; Wang et al. 2021; Piliarová et al. 2019). However, certain inocula reduced the efficacy of OWP in enhancing grain and haulm yields to a lesser extent. This may be attributed, on the one hand, to a negative interaction between specific microbial communities in the soil–OWP–MI system, in particular their competition for certain nutrient resources to the detriment of the plant (Das et al. 2022; Joniec and Furczak 2012). On the other hand, BM also contributes significant quantities of organic matter with a higher C/N ratio than that of OWPs (Table 1), which may slow the overall mineralization process, particularly during season 1.

The highest grain and haulm yields were observed in season 2. The largest difference was observed for grains, with an increase of 774% during season 2. These results can be attributed to diseases (fusariosis) and pests (aphids, bugs, and millipedes) that led to flower drop and pod destruction in season 1 (Image S2). However, the combined influence of the season 1 carryover effect and repeated application in the season 2 trial appeared to positively influence grain and haulm yields. Indeed, the accumulation of nutrients and BMs could lead to better plant nutrition in season 2, thereby increasing yield (Freitas et al. 2025; Olsen et al. 2015). The highest grain yields (3.64 ± 0.39 t ha^−1^) were obtained with PL_BM in season 2, while the lowest grain yields (0.11 ± 0.01 t ha^−1^) were obtained with Fe_Zn in season 1. These grain yields differed from those reported by ISRA with the same variety: 2.9 and 0.63 t ha^−1^ with and without mineral fertilizer, respectively. These differences in “Thieye” grain yields can be explained by differences in the nature, composition, and dose of fertilizer applied, as well as the conditions of the growing environment (physicochemical and biological properties of the soil, in particular).

### 4.2 OWPs and MI Affect Fe Uptake

OWPs and MI affected grain and haulm Fe concentrations in approximately half of all cases and grain and haulm Fe yield in most cases compared with those in the control. We observed significant increases in Fe concentrations (up to +48% in grains and +259% in haulms) and Fe yields (up to +225% in grains and +828% in haulms).

To date, few studies have been conducted on the Fe biofortification of the stems or leaves of plants with multiple edible parts using OWPs. The increase in Fe concentrations in haulms following OWP treatment observed in our study is consistent with the results of several studies on the Fe biofortification of grains or tubers of other crops (Ziarati et al. 2021; Escobedo‐Monge et al. 2020). For instance, Ziarati et al. (2021) showed an increase in Fe concentrations in rice ( *Oryza sativa* ) grains grown on clay‐loamy‐sandy soil fertilized with a mixture of organic waste compared with those in the unfertilized control. These results can be explained by the improved availability of Fe in the soil following the addition of Fe‐rich OWP (Table S2). Shuman (1988) demonstrated that adding organic matter to soil increased the exchangeable and organic Fe and Zn fractions (forms readily available to plants) in the soil. Furthermore, OWPs may specifically affect Fe solubility and bioaccumulation, generally causing variations in the pH and ionic composition of the soil (Escobedo‐Monge et al. 2020). The chemical fortification effect observed with the Fe_Zn treatment on haulm Fe concentrations is consistent with the results of several studies (Dhaliwal et al. 2022; Vaghar et al. 2020; Chen and Barak 1982). For example, Dhaliwal et al. (2022) showed a significant increase in Fe concentrations in soybean ( *Glycine max* ) straw following foliar application of a FeSO_4_·7H_2_O solution. These results can be attributed to Fe uptake into plant leaves being accompanied by translocation throughout the plant. An optimal quantity of absorbed Fe is translocated into grains, and a large quantity remains in the haulms (Vaghar et al. 2020).

MI in combination with OWPs (corresponding to modalities including BMs, MF, or both BM_MF) significantly increased grain Fe concentrations in most cases compared with those in the control or Fe_Zn. These results can be explained by the mineralizing and solubilizing potential of certain microorganisms present in BMs. Indeed, Noumsi‐Foamouhoue et al. (2023) demonstrated that the BMs used in the present study led to carbon overmineralization (up to +41%) from OWPs in a soil–OWP–BM system, which could improve Fe bioavailability in the soil (Dotaniya and Meena 2015). Francis and Dodge (1991) demonstrated that *Clostridium* sp. (Table 2) can reduce Fe^3+^ to Fe^2+^ (the plant‐available form) through enzymatic dissolution or by producing organic acids. Moreover, certain microorganisms such as *Pseudomonas* and *Bacillus* (Table 2) are capable of secreting siderophores, leading to the formation of Fe–siderophore complexes that are easily absorbed by plants (Khan et al. 2019; Wang et al. 1993). MF can also improve plant Fe concentrations. George et al. (1994) specified that, in addition to increasing the absorptive surface of plants, mycorrhized roots can have a higher Fe content than that in non‐mycorrhized roots. Similarly, de Santiago et al. (2011) observed a positive effect of *Trichoderma asperellum* strain T34 on Fe nutrition in wheat. Thus, the greater efficacy of SS_BM_MF compared with that of SS_BM or SS_MF in enhancing haulm Fe concentrations could result from synergy between the microbial communities of BMs and *R. irregularis* . The grain Fe concentrations obtained in our study reached 61.5 mg kg^−1^ with SS_MF, which is higher than those obtained in agrobiofortified rice grains (42.18 mg kg^−1^) reported by Ziarati et al. (2021) using OWPs. This is coherent with the hypothesis that a combination of OWPs and MI would, in most cases, improve the efficacy of OWPs in enhancing Fe concentrations in cowpea grains.

In the present study, grain Fe concentrations decreased by only 8% in season 2, despite an increase in grain yield in season 2 of +774% compared with that in season 1. However, no significant difference was observed between the Fe concentrations in haulms in seasons 1 and 2, despite a +33% increase in haulm yield in season 2. These results indicated a lower dilution of Fe concentrations in grains and none in haulms during season 2. This contrasts with the significant decrease in macro‐ and micronutrient concentrations in certain plant organs generally observed with increased biomass. For instance, the dilution curves established by Cruz and Guillaume (1999) showed a reduction in N, P, and K concentrations in sugarcane ( *Saccharum officinarum* ) leaves alongside an increase in dry biomass expressed in t ha^−1^. Similarly, Pouzet et al. (1999) reported a decrease in Mn concentrations in sugarcane stalks with an increase in dry biomass. These findings further support the hypothesis of a positive carryover effect from seasons 1 to 2, particularly through the accumulation of nutrients and BMs that are likely to enhance Fe availability in the soil.

Fe accumulates in the soil, as evidenced by the large difference between the total quantities of Fe supplied and extracted by cowpeas (grains + haulms) (Table S3). PL and SS supplied 25.7 and 192.14 kg ha^−1^ of Fe, respectively (Table S2). Of these amounts, cowpea extracted 5.03 (19.58%) and 6.29 kg ha^−1^ (3.27%) of Fe from PL and SS, respectively. Consequently, SS left more residual Fe in the soil (185.85 kg ha^−1^) than did PL (20.67 kg ha^−1^). Therefore, monitoring successive OWP inputs (especially SS) over the long term in the same production system is necessary. Although Fe is a micronutrient, its high concentration in the soil (> 800 ppm) could lead to Fe toxicity and nutrient absorption imbalances, particularly for Zn and K (Hasnine et al. 2017; Sahrawat 2005).

### 4.3 OWPs and MI Affect Zn Uptake

OWPs and MI influenced Zn uptake in most cases. We observed a significant increase in Zn concentrations in almost all combinations and plant parts during season 1, but only in three modalities in grains during season 2 (up to +28% in grains and +265% in haulms) and a significant increase in Zn yields in almost all combinations, in grains and haulms, during season 2 (up to +190% in grains and +959% in haulms).

The increase in Zn concentrations in grains and haulms with the addition of OWPs observed in our study is consistent with the results of other studies (Fassinou Hotegni et al. 2024; Ramaiyan et al. 2023; Khalid et al. 2022). Khalid et al. (2022) reported a significant increase in Zn concentrations in the stems and grains of wheat grown on clay‐loam soil fertilized with poultry, sheep, or farmyard manure compost. These results can be explained by the improved Zn availability in the soil following the application of Zn‐rich OWPs (Table S2). Moreover, the significant amount of Zn applied with BMs (Table S2) should also improve Zn availability in treatments involving BMs. Increased Zn availability associated with soil organic matter has been reported by Hafeez et al. (2013). Organic matter promotes Zn solubilization by forming organometallic complexes capable of increasing Zn availability in plant roots. Organic matter also improves soil structure and increases water retention and CEC (Carey et al. 2025; Campdelacreu Rocabruna et al. 2024). This increases the mobility of nutrients, particularly Zn, and facilitates their absorption. The chemical fortification effect observed with the Fe_Zn treatment on Zn concentrations in grains and haulms is comparable to that obtained with the application of a ZnSO_4_·7H_2_O solution in other contexts (Manzeke et al. 2017; Ram et al. 2015; Dhaliwal et al. 2009). Applying Zn‐enriched solutions facilitates Zn mobility in the phloem and translocation in developing grains (Zuchi et al. 2012). Although the highest Zn concentration in grains was obtained with Fe_Zn, the OWP–MI combinations were generally more effective, particularly in increasing Zn yields in grains and haulms.

MI (BM or MF or BM_MF) improved the efficacy of OWPs in enhancing haulm Zn yield, particularly during season 2. The involvement of certain microbial groups or genera present in the BMs used as a Zn source by plants has been previously reported. Prajapati et al. (2021) showed that increased root Zn uptake and concentration in rice haulms are linked to its solubilization by *Pantoea* (Table 2). Khalid et al. (2022) concluded that the increased Zn concentrations observed in wheat grains and straws are linked to the mineralization of compost applied to the soil and Zn solubilization by a combination of microorganisms, including *Sphingobacterium*, *Pseudomonas*, and *Bacillus* (Table 2). Kamran et al. (2017) showed that an increase in Zn concentrations in wheat grains and straw was linked to Zn solubilization by *Enterobacter*, *Rhizobium*, *Pseudomonas*, and *Pantoea* (Table 2). Some of these microorganisms are capable of improving root Zn uptake via the production of siderophores (*Pseudomonas* and *Enterobacter*) or the increased regulation of Fe and Zn transporters (*Enterobacter*) (Singh and Prasanna 2020). Furthermore, Watts‐Williams and Cavagnaro (2018) showed that the positive effects of *R. irregularis* on Zn concentrations in barley ( *Hordeum vulgare* ) stems and grains were associated with the upregulation of Zn transporter genes in the roots. Additionally, mycorrhizal inoculation with *Glomus mosseae* positively influenced Zn and Fe concentrations in millet biomass (Founoune‐Mboup et al. 2024).

In our study, Zn concentrations in grains increased by +220% in season 2, despite an increase in grain yield of +774% in season 2 compared with that in season 1. Contrastingly, haulm Zn concentrations increased by +38% in season 2, despite an increase in haulm yield of +33% in season 2. These results show a positive correlation between grain and haulm yields and Zn concentrations. This contrasts with the trend reported in literature concerning nutrient dilution. For example, Pouzet et al. (1999) observed a significant decrease in sugarcane stalk Zn concentrations concomitant with an increase in dry biomass. In our study, nutrient and BM accumulation in the soil through repeated application of OWPs and MI significantly improved the phytoavailability of Zn and consequently influenced its translocation to various plant organs.

Zn accumulates in the soil, as evidenced by the substantial disparity between the total quantities of Zn supplied and extracted by cowpea (grains + haulms) (Table S3). PL and SS supplied 1.34 and 4.44 kg ha^−1^ of Zn, respectively (Table S2). Of these quantities, cowpea extracted 2.55 Zn kg ha^−1^ (190%) from PL and 3.3 kg Zn ha^−1^ (74.32%) from SS, implying that SS leaves more Zn in the soil (1.14 kg Zn ha^−1^) than does PL (−1.21 kg Zn ha^−1^). Therefore, long‐term management of successive SS inputs is essential within the same production system. Although Zn is an essential micronutrient for plants, excessive soil concentrations (> 500 ppm) can render it a pollutant, posing toxicity risks to microorganisms, plants, and fauna (Hasnine et al. 2017).

## 5 Conclusion

Applying OWPs in combination with MI resulted in significant increases in Fe and Zn concentrations in cowpea haulms and grains. These findings validate our initial hypotheses: (1) the potential of OWP‐derived micronutrients to enhance the agrobiofortification of cowpeas, and (2) the added value of micronutrient gains with MI augmentation. The combination of SS (6.11 t ha^−1^), BM (15 t ha^−1^), and MF (1.88 t ha^−1^) is recommended for Fe and Zn agrobiofortification of cowpea haulms and grains. Further studies should focus on: (1) evaluating the effects of combined applications of OWPs and MI on Fe and Zn concentrations in cowpea pods, and (2) assessing the environmental impact of the established system by analyzing the chemical composition and microbial communities of the soil. In addition, the effectiveness of the combination of OWPs and MI on Fe and Zn agrobiofortification should be verified in other widely consumed local crops, such as the orange‐fleshed sweet potato.

## Author Contributions

Conceptualization: Emmanuel Noumsi‐Foamouhoue, Samuel Legros, Paula Fernandes, and Hassna Founoune‐Mboup, and Jean‐Michel Médoc. Funding acquisition: Jean‐Michel Médoc. Methodology: Emmanuel Noumsi‐Foamouhoue, Samuel Legros, Paula Fernandes, Hassna Founoune‐Mboup, Bassirou Diallo, Komi Assigbetsé, and Jean‐Michel Médoc. Supervision: Samuel Legros, Paula Fernandes, Aboubacry Kane, Frédéric Feder, and Jean‐Michel Médoc. Data curation and visualization: Emmanuel Noumsi‐Foamouhoue. Writing – original draft: Emmanuel Noumsi‐Foamouhoue. Writing – review and editing: Emmanuel Noumsi‐Foamouhoue, Samuel Legros, Paula Fernandes, Hassna Founoune‐Mboup, Bassirou Diallo, Komi Assigbetsé, Aboubacry Kane, Frédéric Feder, and Jean‐Michel Médoc. All authors have read and agreed to the published version of the manuscript.

## Funding

This research was funded by the African Union Commission and the European Union Commission (grant number AURG‐II‐2‐110‐2018). Additional support for the valorization and dissemination of results was provided by the World Bank through the NBS Invest Fund (Accelerating Nature‐Based Solutions in Least Developed Countries), within the framework of the ASA Reflection and Knowledge project (contract number 0002010826).

## Ethics Statement

The authors have nothing to report.

## Consent

The authors have nothing to report.

## Conflicts of Interest

The authors declare no conflicts of interest.

## Supporting information


**Appendix S1:** fsn371375‐sup‐0001‐Supinfo.docx.

## Data Availability

The data that support the findings of this study are available from the corresponding author upon reasonable request.
