# Supplementary material for: Synergistic Effect of Organic Waste Products and Microbial Inocula on Iron and Zinc Biofortification in Cowpea [Vigna unguiculata (L.) Walp.]
Source: Food Sci Nutr. 2026 Jan 5;14(1):e71375. doi: 10.1002/fsn3.71375 (PMC12771656; doi:10.1002/fsn3.71375)
Supplement: Supplementary file 1 — Appendix S1: fsn371375‐sup‐0001‐Supinfo.docx. [file FSN3-14-e71375-s001.docx]

**Supplementary Material**

**Synergistic Effect of Organic Waste Products and Microbial Inocula on Iron and Zinc Biofortification in Cowpea [*Vigna unguiculata* (L.) Walp.]**

Emmanuel Noumsi-Foamouhoue^1,2,3*^, Samuel Legros^1,3,4^, Paula Fernandes^2,5,6^, Hassna Founoune-Mboup^8^, Bassirou Diallo^8^, Komi Assigbetsé^2,7^, Aboubacry Kane^9,10^, Frédéric Feder^1,3^, Jean-Michel Médoc^1,2,3*^

^1^CIRAD, UPR Recyclage et Risque, F-34398 Montpellier, France

^2^Laboratoire Mixte International IESOL, ISRA-IRD Bel-Air Center, Dakar BP 1386, Senegal

^3^Recyclage et Risque, Université de Montpellier, Montpellier, France

^4^CIRAD, UPR Recyclage et Risque, Saint-Denis, La Réunion F-97743, France

^5^UPR HortSys, CIRAD, F-34398 Montpellier, France

^6^HortSys, Université de Montpellier, Montpellier, France

^7^Institut de Recherche pour le Développement (IRD), F-34394 Montpellier, France

^8^Laboratoire National de Recherches sur les Productions Végétales (LNRPV), Institut Sénégalais de Recherche Agriocole (ISRA), Dakar, Senegal

^9^FST, Département de Biologie Végétale, UCAD, Dakar BP 1386, Senegal

^10^Laboratoire Commun de Microbiologie (LCM), IRD-ISRA-UCAD Bel-Air Center, Dakar BP 1386, Senegal

***Correspondence**: emmanuelnofoma@yahoo.com or [emmanuel.noumsi_foamouhoue@cirad.fr](mailto:emmanuel.noumsi_foamouhoue@cirad.fr) (ENF); [jean-michel.medoc@cirad.fr](mailto:jean-michel.medoc@cirad.fr) (JMM)

Supplementary Tables

**Table S1**

Quantities of N, P, and K supplied by OWP and chemical supplements.

|  | | | N | P | K | Calculation | References |
| --- | --- | --- | --- | --- | --- | --- | --- |
| Recommended dose of element (kg ha^−1^) | | | 0 | 30.562 | 66.24 | (1) | (Gret et al., 2002) |
| Fertilizer equivalence coefficient | PL | | 0.6 | 0.65 | 1 | (2) | (Leclerc, 2001) |
|  | SS | | 0.45 | 0.6 | 1 |  |  |
| Total concentration of the element (kg kg^−1^ DM) | PL | | 0.065 | 0.012 | 0.032 | (3) |  |
|  | SS | | 0.025 | 0.009 | 0.002 |  |  |
| Effective concentration of the element (kg kg^−1^ DM) | PL | | 0.039 | 0.008 | 0.032 | (4) = (3) × (2) |  |
|  | SS | | 0.011 | 0.005 | 0.002 |  |  |
| Theoretical quantity of OWP (t DM ha^−1^) | PL | | 0 | 3.82 | 2.07 | (5) = (1) ÷ [1000 × (4)] |  |
|  | SS | | 0 | 6.11 | 33.12 |  |  |
| Actual quantity of OWP (t DM ha^−1^) | PL | 2.07 |  | | | (6) |  |
|  | SS | 6.11 |  | | |  |  |
| Actual quantity of element supplied with OWP (kg ha^−1^) | PL | | 80.73 | 16.56 | 66.24 | (7) = (4) × [1000 × (6)] |  |
|  | SS | | 67.21 | 30.55 | 12.22 |  |  |
| Quantity of element supplied with mineral supplements (kg ha^−1^) | PL | | 0 | 14 | 0 | (8) = [(5 − 6) × (1)] ÷ (5) |  |
|  | SS | | 0 | 0 | 54.02 |  |  |
| Total quantity of element supplied (kg ha^−1^) | PL | | 80.73 | 30.56 | 66.24 | (7) + (8) |  |
|  | SS | | 67.21 | 30.55 | 66.24 |  |  |

OWP (organic waste product), PL (poultry litter), SS (sewage sludge), N (nitrogen), P (phosphorus), K (potassium), DM (Dry Matter).

**Table S2**

Quantities of iron and zinc provided by OWP.

|  |  | | Fe | Zn | Calculation |
| --- | --- | --- | --- | --- | --- |
| Total concentration of the element (mg kg^−1^ MS) | PL | | 6210 | 326 | (1) |
|  | SS | | 15724 | 364 |  |
|  | BM | | 4.34 | 20 |  |
| Quantity of OWP and BM applied (t MS ha^−1^) | PL | 2.07 |  | | (2) |
|  | SS | 6.11 |  | |  |
|  | BM | 15 |  | |  |
| Total concentration of the element (kg ha^−1^) | PL | | 12.85 | 0.67 | (3) = [(1) × (2)] ÷ 1000 |
|  | SS | | 96.07 | 2.22 |  |
|  | BM | | 0.07 | 0.3 |  |

OWP (organic waste product), PL (poultry litter), SS (sewage sludge), BM (local beneficial microorganisms), Fe (iron), Zn (zinc), DM (Dry Matter).

**Table S3**

Iron and zinc extraction rates by cowpea (grains and haulms).

|  | |  |  | Supplied quantities (kg/ha) | Extracted quantities (kg/ha) | Extraction rate (%) | Residual quantities in soil (kg/ha) |
| --- | --- | --- | --- | --- | --- | --- | --- |
|  |  |  |  |  |  |  |  |
| Iron | PL | Season 1 | Grains | 12.85 | 0.017 ± 0.004 | 15.89 ± 4.83 | 10.81 ± 0.62 |
|  |  |  | Haulms |  | 2.026 ± 0.620 |  |  |
|  |  | Season 2 | Grains | 12.85 | 0.145 ± 0.049 | 23.26 ± 7.57 | 9.86 ± 0.97 |
|  |  |  | Haulms |  | 2.845 ± 0.971 |  |  |
|  |  | Sum |  | 25.7 | 5.033 ± 1.644 | 19.58 ± 6.40 | 20.67 ± 1.64 |
|  | SS | Season 1 | Grains | 96.07 | 0.021 ± 0.009 | 3.19 ± 1.58 | 93.00 ± 1.52 |
|  |  |  | Haulms |  | 3.046 ± 1.520 |  |  |
|  |  | Season 2 | Grains | 96.07 | 0.148 ± 0.048 | 3.35 ± 0.79 | 92.85 ± 0.76 |
|  |  |  | Haulms |  | 3.077 ± 0.754 |  |  |
|  |  | Sum |  | 192.14 | 6.292 ± 2.331 | 3.27 ± 1.21 | 185.85 ± 2.33 |
| Zinc | PL | Season 1 | Grains | 0.67 | 0.004 ± 0.001 | 130.15 ± 22.84 | −0.20 ± 0.15 |
|  |  |  | Haulms |  | 0.868 ± 0.153 |  |  |
|  |  | Season 2 | Grains | 0.67 | 0.152 ± 0.049 | 250.29 ± 97.14 | −1.01 ± 0.65 |
|  |  |  | Haulms |  | 1.525 ± 0.649 |  |  |
|  |  | Sum |  | 1.34 | 2.549 ± 0.852 | 190.22 ± 63.58 | −1.21 ± 0.85 |
|  | SS | Season 1 | Grains | 2.22 | 0.004 ± 0.001 | 44.05 ± 14.82 | 1.24 ± 0.33 |
|  |  |  | Haulms |  | 0.974 ± 0.329 |  |  |
|  |  | Season 2 | Grains | 2.22 | 0.124 ± 0.038 | 104.59 ± 24.11 | −0.10 ± 0.54 |
|  |  |  | Haulms |  | 2.198 ± 0.534 |  |  |
|  |  | Sum |  | 4.44 | 3.300 ± 0.902 | 74.32 ± 20.32 | 1.14 ± 0.90 |

PL (poultry litter), SS (sewage sludge).

Supplementary Figures


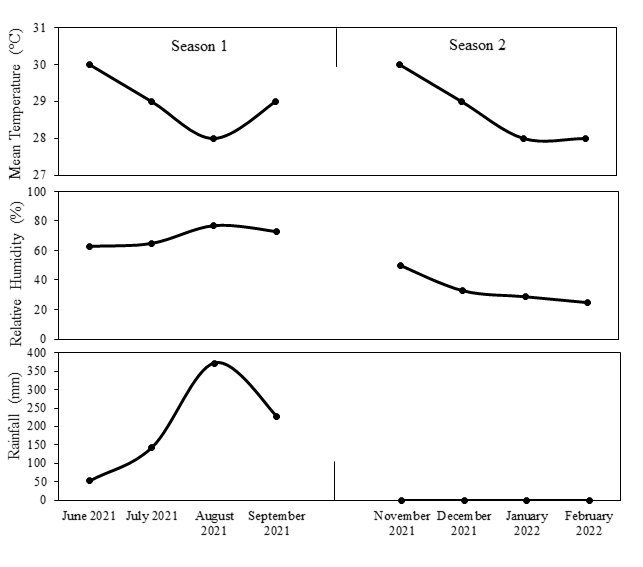


**Figure S1.** Meteorological data for the study site (data source: SDDR, 2022).

Supplementary images

**Image S1**


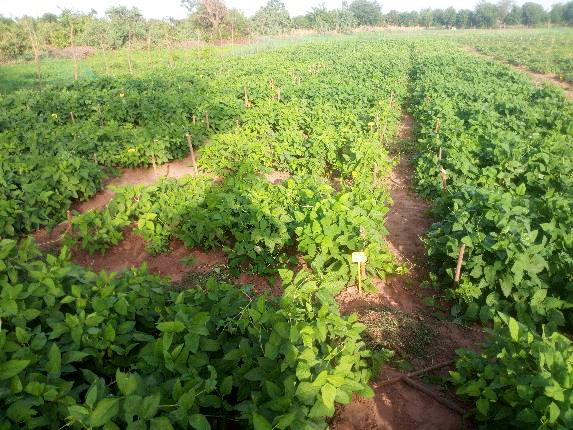


**Image S1.** Total cowpea plot after weeding and hoeing. Season 1. ISRA experimental station. Nioro du Rip. Senegal.

**Image S2**

**Image S2.** Presence of fusariosis (a), stink bug (b), and millipede (c) on cowpea plants during season 1. ISRA experimental station. Nioro du Rip. Senegal.

**References**

Gret. Cirad. Ministère français des Affaires étrangères. 2002. Mémento de l’agronome. Montpellier : CIRAD-GRET. 1691 p.

Leclerc. B.. 2001. Guide des matières organiques. Tome 2. ed. Institut Technique de l’Agriculture Biologique. Paris CEDEX 12.

SDDR. 2022. Services départementaux de développement rural. Nioro du Rip. Sénégal.
